# Follicular homocysteine as a marker of oocyte quality in PCOS and the role of micronutrients

**DOI:** 10.1007/s10815-023-02847-3

**Published:** 2023-06-10

**Authors:** Tansu Kucuk, Pınar Erol Horozal, Asena Karakulak, Emel Timucin, Maurizio Dattilo

**Affiliations:** 1 Acibadem Fulya Hospital IVF Center, Istanbul, Turkey; 2 Partogen, Istanbul, Turkey; 3 Department of Biostatistics and Medical Informatics, School of Medicine, Acibadem University, Istanbul, Turkey; 4 Parthenogen, R&D Department, Lugano, Switzerland

**Keywords:** Homocysteine, PCOS, Follicular fluid, Blastocyst rate, Micronutrients

## Abstract

**Purpose:**

Does follicular homocysteine predict the reproductive potential of oocytes following FSH stimulation in PCOS women? Can it be modulated by dietary interventions?

**Methods:**

This was a prospective, randomized, interventional clinical study. Forty-eight PCOS women undergoing in vitro fertilization at a private fertility clinic were randomized for a dietary supplementation providing micronutrients involved in homocysteine clearance or no treatment. The supplement was assumed 2 months before stimulation until pick-up day. Monofollicular fluids were collected and frozen. After embryo transfer, the fluids from the follicles generating the transferred embryos were thawed and analyzed.

**Results:**

Follicular homocysteine showed a negative correlation with clinical pregnancy both in the whole population (*r* =  − 0.298; *p* = 0.041) and in controls (*r* =  − 0.447, *p* = 0.053). The support achieved a non-significantly lower concentration of follicular homocysteine (median [IQR]–7.6 [13.2] vs 24.3 [22.9]). Supplemented patients required far less FSH for stimulation (1650 [325] vs 2250 [337], *p* = 0.00002) with no differences in the number of oocytes collected, MII rate, and fertilization rate. Supplemented patients enjoyed higher blastocyst rate (55% [20.5] vs 32% [16.5]; *p* = 0.0009) and a trend for improved implantation rate (64% vs 32%; *p* = 0.0606). Clinical pregnancy rates were 58% vs 33% in controls (*p* = ns).

**Conclusion:**

Follicular homocysteine is a suitable reporter that might be investigated as a tool for oocyte-embryo selection. A diet enriched with methyl donors may be useful in PCOS and supplements may also help. These findings may be also true for non-PCOS women, which warrants investigation.

The study was approved by the Acibadem University Research Ethics Committee (2017–3-42). Clinical trial retrospective registration number ISRCTN55983518.

## Introduction

Homocysteine (Hcy) is a non-proteogenic amino acid resulting from (adenosyl)methionine de-methylation in transmethylation reactions. Due to the wide involvement of carbon unit trafficking in animal metabolism, Hcy is massively and continuously produced within cells with one-fifth to one-sixth of the total Hcy pool renovated every hour [1]. It is continuously re-cycled by either re-methylation to methionine, thus restoring the methylation capacity, or transsulfuration to cysteine, which feeds the synthesis of the universal cellular antioxidant glutathione (GSH) and of the reducing gasotransmitter hydrogen sulfide (H_2_S), within the so-called one carbon metabolism pathway (OCM). Any impairment of Hcy re-cycling results in increased Hcy concentration in blood, i.e., hyperhomocysteinemia (HHcy). Besides being a reliable reporter of metabolic dysfunction in the OCM, HHcy is also potentially deleterious because it acts as a powerful inhibitor of methylations and as a dangerous pro-oxidant [2], thus hampering the same pathways it belongs to. HHcy has been linked to a variety of pathological conditions including atherosclerosis, myocardial infarction, ischemic stroke, neurodegeneration, osteoporosis, cancer, and thrombotic events [3, 4].

The OCM is fundamental for gametogenesis and embryogenesis. Methylations are necessary to ensure DNA methylation as part of the epigenetic programming as well as for key molecular syntheses and in general for tissue growth and differentiation. The endogenous antioxidant system supported by GSH is in turn necessary to neutralize the reactive oxygen species (ROS) generated in mitochondria, thus protecting the gametes and embryo DNA from oxidative degeneration. Accordingly, HHcy has been linked to defective gametogenesis in both sexes and to couple infertility [5] as well as to a variety of pregnancy complications [6].

Polycystic ovary syndrome (PCOS) is an endocrine disorder occurring in up to 20% of women in reproductive age [7]. The clinical manifestations are variable; however, the association with androgen excess, insulin resistance, obesity, and metabolic syndrome points to a metabolic pathogenesis. PCOS women have been reported to suffer HHcy [8], although the finding is more common in those with insulin resistance and androgen excess as well as it is difficult to exclude a negative effect on Hcy metabolism from the treatment with metformin and oral contraceptives that these women often assume [9].

HHcy has been shown to mark a worst reproductive prognosis in PCOS women undergoing intra-uterine insemination (IUI). In a IUI trial [10], the clinical pregnancy rate of PCOS women with a serum Hcy concentration above 15 µmol/l was just 14.29% compared to 37.88% in those with Hcy below 15 µmol/l (*p* = 0.044). However, most of the Hcy recycling job is exerted by the liver and fasting blood Hcy concentration may not directly report what happens in ovarian follicles. Local imbalances due to increased demand could occur within follicles, even more if the patient undergoes ovarian hyperstimulation to induce multiple ovulation for in-vitro fertilization. Indeed, the maturing follicle has an increased burden of methylations leading to accumulation of Hcy that should be re-cycled internally while the availability of re-cycling substrates, e.g. folates, remains the same [11]. This may result in relative metabolic imbalances with Hcy accumulation in follicles even without a measurable increase in blood. Indeed, in women undergoing COH, high Hcy in the fluid of the leading follicle correlated with poor embryo quality [12]. Accordingly, Hcy concentration in follicular fluid could be a better marker of the metabolic competence of a given follicle.

Berker et al. (2009) found that a higher amount of Hcy in follicular fluid of PCOS women undergoing oocyte pick-up following FSH stimulation sharply marked lower oocyte quality, fertilization rates, embryo quality, and pregnancy rates with no pregnancies occurring in women whose sampled follicles contained more than 8 μmolar Hcy [13]. Interestingly, the blood Hcy of the same patients was normal (11.7 ± 2.9 μmol) suggesting the occurrence of a follicle-specific impairment of Hcy metabolism in PCOS and a possible role of Hcy in PCOS subfertility also in women with normal Hcy blood values. These findings prompted follicular homocysteine as a possible quality marker for oocyte selection in assisted reproduction but the data from Berker et al. (2009) [13] could not address the question because there was no overlapping identity between the follicles sampled for follicular Hcy and the oocytes eventually used to perform the (multiple) embryo transfers.

**Fig. 1:**
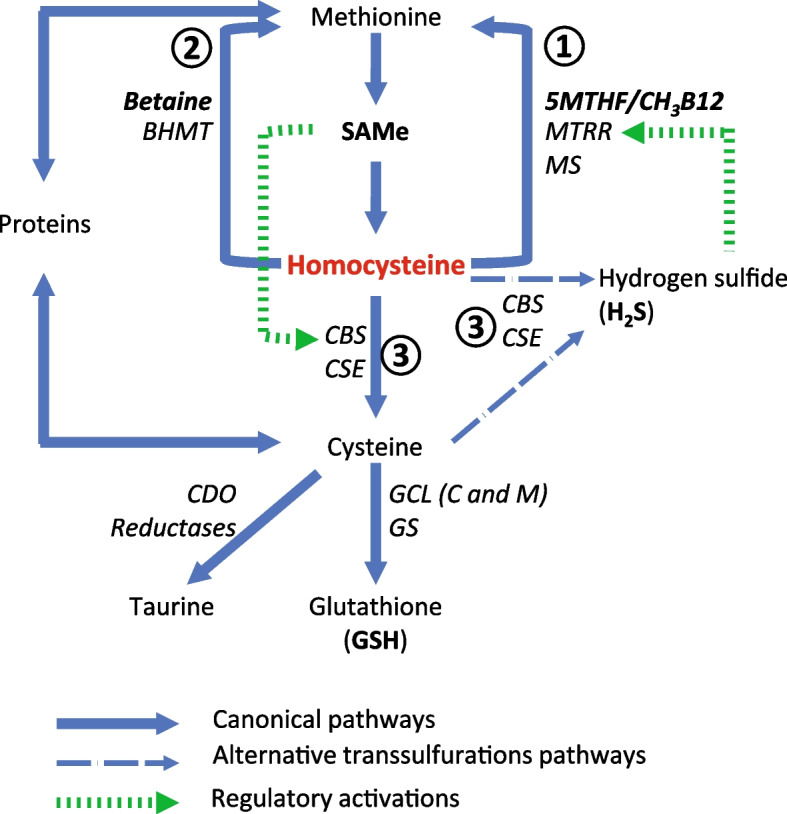
Pathways for homocysteine removal. Any excess of Hcy is toxic and must be eliminated. On the other side, homocysteine is the substrate for other syntheses and a balanced partitioning among the available pathways is essential to avoid metabolic derangements. Hcy can be re-methylated to methionine/SAMe using the methyl group from 5MTHF/CH3B12 (①) under the sequential actions of MTRR and MS. In alternative, it can be re-methylated by BHMT using the methyl group from betaine (②), which happens mainly in the liver. Finally, Hcy can be transsulfurated to cysteine by the sequential action of CBS and CSE (③). The latter enzymes exert an ambiguous recognition of their substrates and may work in a reverse manner (alternative transsulfurations) using cysteine and Hcy to release the reducing gasotransmitter Hydrogen sulfide (H_2_S). Any cysteine in excess on the demand for protein synthesis is used to generate the universal reducing agent GSH, feeding the antioxidant system. In case of further excess of cysteine, the enzyme CDO undergoes extreme up-regulation to consume it in reducing reactions with taurine as the final product. CBS and CSE act as the main regulators of Hcy partitioning. When SAMe is in excess, it operates an allosteric activation of CBS with a fivefold increase of activity [27] so that more of Hcy is converted to cysteine to balance the excess of SAMe. On the other side, several studies have reported up-regulation of methylations when antioxidant power/GSH is in excess, but no mechanism has been described. However, H_2_S generated from the excess of Hcy entering the CBS/CSE alternative pathways has been proposed as an activator of MTRR [28], thus enhancing Hcy re-methylation by MS. BHMT = betaine homocysteine methyl transferase; CBS = cystathionine beta synthase; CDO = cysteine di-oxygenase; CSE = cystathionine γ-lyase; GCL = glutamate cysteine ligase with catalytic (C) and regulatory (M) subunits; GS = glutathione synthase; MS = methionine synthase; MTRR = methionine synthase reductase

Excess of Hcy is removed by three alternative pathways (Fig. 1): re-methylation to methionine by a methyl group donated by either (1) methyltetrahydrofolate/methylcobalamin or (2) betaine and (3) Hcy transsulfuration to cysteine, which is used for protein synthesis or to release the universal cellular antioxidant GSH [14]. A combined micronutrient supplementation supporting both the re-methylation (folates, B2, B3, B6, B12, betaine, and zinc) and the transsulfuration (B6, cysteines, and zinc) of Hcy (Impryl, Parthenogen, Switzerland) had been already shown to be very efficient in decreasing blood fasting Hcy in young PCOS women with some decrease in every treated patient [15], although the effect of the supplementation on their reproductive competence had not been investigated. The same supplementation has the potential to lower the Hcy level also in the follicular fluid, which may result in improved fertility, but such a potential has not been tested so far.

The present study was intended to preliminarily explore the role of follicular Hcy in predicting the reproductive potential of oocytes, i.e., their ability to generate a viable blastocyst, in assisted reproduction cycles and the ability of a micronutrient support to decrease Hcy concentration in the follicular fluid of PCOS women undergoing controlled ovarian hyperstimulation (COH) for assisted reproduction purposes.

## Methods

### Study design, inclusion criteria, and randomization

This was a prospective, randomized, parallel group, open label, controlled versus no treatment clinical study. The study was approved by the Acibadem University Research Ethics Committee. Clinical trial retrospective registration number ISRCTN55983518.

The patients were enrolled between April 2017 and February 2022. The study enrolled women aged more than 18 with primary infertility for at least 1 year, diagnosed PCOS according to the Rotterdam criteria [16] and addressed to their first COH for assisted reproduction purposes. Reasons for exclusion were as follows: other known reasons for infertility; ongoing treatment with sugar lowering agents, anti-hypertensives, or hormones; systemic or endocrine diseases; and assumption of any dietary supplement in the week before enrolment or need to assume supplements during the study period. After a check of the inclusion/exclusion criteria, a written informed consent was obtained and a randomization position was assigned according to a computer-generated balanced block list, block size 4.

### Study procedures

The patients randomized for the treatment arm also received instructions on how to assume the supplement and how to report on adverse events. They assumed one tablet per day of a supplement containing essential or conditionally essential micronutrients in support to the OCM including: betaine 200 mg, l-cystine 200 mg, chelated zinc 10 mg, niacin (vit. B3) 16 mg, pyridoxine (vit. B6) 1.4 mg, riboflavin (vit. B2) 1.4 mg, 5-methyl-tetrahydrofolate (5MTHF) 400 μg, and methylcobalamin (CH_3_B12) 2.5 μg (Impryl, Parthenogen, Switzerland). They consumed the supplement since at least 2 months before ovarian stimulation and until the oocyte pick-up day.

All patients underwent COH according to the standard practices of the study site for the same patients without any study-intended adjustments. Briefly, patients received 150 IU of rFSH daily until day 5; thereafter, the FSH dose was adjusted according to the patient’s response. When the leading group of follicles reached 13 mm, a GnRH antagonist was commenced to inhibit premature LH surge. Once at least 3 follicles reached 18 mm diameter, a bolus of rhCG was injected to induce final maturation. The oocyte pick-up was performed 35–36 h post hCG injection by US-guided transvaginal needle aspiration. The fluid contained in each punctured follicle was collected immediately after aspiration and placed on ice until centrifugation to eliminate any cells and frozen at − 20. Mature oocytes were injected with the partner sperm and fertilization was confirmed by the appearance of two pronuclei. The zygotes were cultured up to blastocyst stage. The best embryo based on morphological score was selected for the fresh embryo transfer. All patients received luteal support with micronized progesterone, 600 mg per day, vaginal route.

The follicular fluid samples corresponding to the oocytes that generated the blastocysts selected for transfer were thawed and analyzed for Hcy content. The concentration of Hcy in follicular fluids was tested by a commercially available ELISA kit (Sunred Bio, China) with a sensitivity range of 0 to 155 μmol/L, an intra-assay variability < 10%, and inter-assay variability < 12%. The follicular fluid samples of concern were thawed and tested at a single laboratory all together after the completion of the follow-up of the last enrolled patient.

### Clinical endpoints

Biochemical pregnancy rates were calculated as the rate of patients with positive beta-hCG 7 days post embryo transfer out of the number of patients receiving an embryo transfer. Implantation rates were calculated as the number of gestational sacs observed at vaginal ultrasound 7 weeks after transfer out of the number of transferred embryos. Clinical pregnancy rates were calculated as the rate of patients with fetus heart activity beyond 7 weeks of gestation out of the number of patients receiving an embryo transfer.

### Statistical analysis

This was a pilot study and, in the absence of previous data to build a statistical hypothesis, the sample was not statistically sized. The inclusion of 40 patients, 20 per group, was believed to be a clinically significant sample allowing to calculate the sample size for any future larger studies. Assuming a drop-out rate post-randomization of 20%, a total of 48 patients (24 per group) was randomized.

Categorical data were summarized by absolute frequencies and tested by the chi-square tests with Yates’ correction. Continuous data did not show a normal distribution, therefore they were summarized by median and interquartile range (IQR) and tested by Wilcoxon Rank-sum tests. Statistical computations and visualizations were carried out by R-studio (2022.07.2), which is the integrated development environment of R. In all analyses, a *p* value < 0.05 was considered significant.

## Results

Five patients, all from the intervention group, withdrew their consent to the study post randomization so that 43 patients completed the study: 19 in the intervention group and 24 in the control group. None of the patients consuming the supplement reported adverse events.

The demographic and baseline characteristics of the two groups of patients are reported in Table 1. The groups were balanced for all the tested parameters apart free testosterone that was significantly higher in the intervention group.

**Table 1:** Demographic and baseline characteristics of patients

	Controls (*n* = 24)	Treatment (*n* = 19)	*W*/*Χ*^2^*	*p* value	Total (*n* = 43)
Age	37.5 (7.75)	36 (2.5)	261.5	0.418	37 (5.5)
BMI	23.25 (3.9)	23.8 (4.25)	202	0.5327	23.7 (4.45)
Smoking, n (%)	14 (58)	9 (47)	0.167*	0.684	23 (53)
FSH	6.35 (1.9)	6.02 (2.58)	216	0.7784	6.3 (2.25)
LH	8.7 (1.275)	8.04 (2.19)	252	0.5651	8.7 (2.29)
AMH	4.95 (2.275)	4.7 (2.9)	234	0.8929	4.8 (2.75)
Estadiol (E_2_)	76.25 (11.075)	73 (15)	254	0.5326	76 (13.7)
Testosterone, total	46.9 (23.25)	54.4 (23.9)	191.5	0.3786	49.5 (26.3)
Testosterone, free	4.5 (3.725)	6.6 (3.05)	134	0.02214	5.5 (3.5)
DHEA	154 (93.1)	151.4 (117.25)	224.5	0.9415	151.4 (111.45)
HOMA	2.05 (0.5)	1.9 (0.6)	239.5	0.7857	2 (0.55)
SHBG	77.25 (48.5)	71.1 (73.45)	238.5	0.8068	77 (55.7)

Continuous data are presented as median (IQR) and analyzed by Wilcoxon rank sum test (W). Categorical data (smoking) are summarized by absolute frequencies and analyzed by chi-square tests (*Χ*^2^*)

The stimulation and clinical outcomes are summarized in Table 2. There were no cases of ovarian hyperstimulation syndrome (OHSS). The patients assuming the micronutrients required significantly less FSH to reach the hCG triggering parameters (1650 IU (325) vs 2250 IU (337); *p* = 0.00002). Such a large difference was not due to the duration of the stimulation, overlapping between groups. Moreover, in spite of the smaller amount of FSH injected, the treatment group achieved a non-significantly higher estradiol concentration (2546 (1002) vs 2089 (1060); *p* = 0.5326). The total number of oocytes collected and their maturation (MII) rate were not different between groups as well as the fertilization rate. However, the zygotes generated by the intervention group showed a significantly improved reproductive potential with a blastocyst rate that was almost double the one achieved in controls (55% vs 32%; *p* = 0.0009).

**Table 2:** Stimulation and clinical outcomes

	Controls *n* = 24	Intervention *n* = 19	*W*/*Χ*^2^*	*p* value	Total *n* = 43
Total FSH	2250 (337.5)	1650 (325)	402	0.00002	1975 (587.5)
Days to hCG	11 (2)	11 (2.5)	193.5	0.3944	11 (2)
PEAK E_2_	2089.5 (1060.5)	2546 (1002)	181	0.2554	2416 (1058)
Oocytes	12.5 (7.5)	15 (7.5)	247	0.6492	14 (7)
MII oocytes	11 (6)	10 (5)	278.5	0.2193	10 (5.5)
MII rate	0.8 (0.117)	0.76 (0.14)	298.5	0.08646	0.77 (0.11)
2PN	9 (3.75)	9 (2.5)	268	0.3286	9 (3)
Fertilization rate	0.82 (0.155)	0.81 (0.165)	228.5	1	0.82 (0.17)
D5 blastocyst	3.5 (2)	4 (2)	164.5	0.1164	4 (3)
Blastocyst rate	0.32 (0.165)	0.55 (0.205)	92	0.0009	0.4 (0.25)
Implantation rate	32%	64%	3.5192*	*0.0606*	47%
Clinical pregnancy rate	33%	58%	1.6937*	0. 1931	44%
Follicular Hcy	24.34 (22.85)	7.62 (13.19)	241	0.6208	10.61 (18.23)

Continuous data are presented as median (IQR) and analyzed by Wilcoxon rank sum test (W). Categorical data (pregnancy rates) are summarized by absolute frequencies and analyzed by chi-square tests with Yates’ correction (*Χ*^2^*)

A positive beta-hCG at 7 days after embryo transfer was recorded in 12 out of 24 patients (50%) in the control group compared to 15 out of 19 patients (79%; *p* = ns) in the group assuming the micronutrients. The 24 patients in the control group received 25 embryo transfers (1 double transfer) resulting in 8 clinical pregnancies whereas the 19 patients in the micronutrient group received 22 embryo transfers (3 double transfers) resulting in 14 clinical pregnancies. The resulting implantation rates per embryo transfer were, respectively, 32% vs 64%, which showed a trend for statistical significance (*p* = 0.0606). The clinical pregnancy rate per patient were 33% (8 out of 24) in the control group and 58% (11 out of 19) in the micronutrients group (*p* = ns).

The follicular concentration of Hcy showed a negative correlation with the clinical pregnancies (*r* =  − 0.298; *p* = 0.041) in the whole patient group and a borderline correlation was also observed when looking at the control group only (*r* =  − 0.447, *p* = 0.053). Such correlation was lost when we considered only the intervention group (*r* =  − 0.369, *p* = 0.84). The concentration of Hcy in the follicular fluids belonging to the follicles maturing the oocytes eventually resulting in a transferred blastocyst was non significantly lower in the micronutrient group (7.62 (13.19) μmol/L vs 24.34 (22.85) μmol/L). However, no pregnancies occurred from follicles whose Hcy was above 10.5 μmol/L apart one patient from the treatment group achieving clinical pregnancy in spite of a higher follicular Hcy.

## Discussion

This was, to our knowledge, the first study assessing the relationship between the concentration of Hcy in the follicular fluid and the development potential in assisted reproduction cycles of the oocyte contained in the same follicle/fluid tested. We selected a population of PCOS patients because they seem to have a specific problem with Hcy [8] and because it was already shown that their follicular Hcy has some predictive value [13]. In our study, follicular Hcy exerted a significant negative correlation with the achievement of a clinical pregnancy. Moreover, out of 22 clinical pregnancies recorded in our patients, only one occurred from an oocyte whose follicular fluid contained more than 10.5 μmol/L of Hcy.

The reasons for an increased circulating Hcy are variable and range from imbalanced diet, poor in leafy greens (shortage of folates) or, opposite, entirely vegetarian (shortage of B12), to a bad fit between the diet and the inherited genetic variants of the concerned metabolic enzymes, in many cases circulating in double digit prevalence, which may generate an increased demand (relative shortage) of several dietary substrates [14]. The individual diet and genetic background are further challenged by the environmental exposures as all known endocrine disruptors [17] and heavy metals [18] specifically interfere with these pathways.

However, although associated to a lower number of retrieved oocytes, blood fasting Hcy is not predictive of assisted reproduction outcomes [19]. Blood Hcy indeed mainly reflects the massive metabolic activity of the liver and provides little information on tissue-specific imbalances, with large variability in Hcy concentration from one organ to another [20]. Thus, issues of increased demand may occur in selected organs at specific time points and generate serious local metabolic imbalances with no detectable changes in circulating levels of Hcy. This is indeed the case of the ovarian follicles during the days of maturation of their oocyte, even more if COH is inducing such stress in multiple follicles at the same time [11]. Even if a single follicle, or several follicles, will be producing very big amounts of Hcy, this release will not be enough to induce blood HHcy, nevertheless it will be enough in the single follicle to cause metabolic failure.

We are now showing that, within assisted reproduction treatments, the detection of Hcy in the follicular fluid is better informative than the blood measurement and exerts a significant negative correlation with the achievement of a clinical pregnancy. The correlation was lost when looking only at the treatment group, likely due to a smaller sample. This vouches for this test as a possible tool for the selection of the best oocytes-embryos to be transferred. Hcy dosing is relatively cheap, it is offered by every clinical laboratory and the test results are usually available within 24 h from sampling, i.e., early enough to be used as an aid in embryo selection. Finally, the test does not expose the patients to increased clinical risk.

Assumed that high Hcy in follicular fluid is not just a reporter but also a cause of damage, it was of relevance to check if dietary manipulations have the potential to improve Hcy metabolism also within the ovarian follicles. To this aim, we used a combination of micronutrients that had already shown high efficacy in reducing blood Hcy in PCOS women with a significant effect also in the subgroup of patients with starting Hcy concentration within the normal limit [15]. In this study, we failed to confirm this effect, likely due to the small sample size. However, the median follicular Hcy concentration of cases was just 30% of that recorded in their controls, which warrants better sized studies to clarify this point.

Supplemented patients required far less FSH to achieve the criteria for hCG triggering. The large difference between the groups is not explained with a different duration of stimulation, which was overlapping between the groups. Moreover, a smaller amount of FSH (− 27%) induced a larger amount of circulating estradiol (+ 22%), which seems to indicate an increased sensitivity to FSH stimulation, i.e., a true change in the behavior of the follicles. It is to be noted that estrogens are a powerful genomic inducer of another product of transsulfurations, hydrogen sulfide (H_2_S) [21], a reducing gas that inhibits Hcy damages in female reproductive tissues [22], and may be involved in the positive effects from the treatment.

In spite of no detectable differences in oocyte number, maturation and fertilization, the zygotes from supplemented follicles resulted in a blastocyst rate that was almost double the one in their controls. Our experimental model does not allow to infer on the reasons for this difference; however, these findings overlap with the one resulting from a recent study based on the use of comparable amounts of the same micronutrients supplemented during the 24-h in vitro maturation of bovine oocytes [23]. Also, in this case, after overlapping maturation and fertilization rate, the supplemented oocytes exerted a double blastocyst rate as compared to their controls (32.5% vs 15.7%; *p* < 0.001). This huge difference was attributed to the strongly enhanced methylation of female pronuclei in supplemented zygotes, which fits with the known metabolic properties of our micronutrients and is a likely explanation also for the present findings. The significant effect on blastocyst development may appear in conflict with no effect on follicular Hcy, showing a large but non-significant reduction. However, this is in agreement with the rationale of our intervention that was aimed at activating the whole pathway providing methylations, nucleic acid synthesis, and redox balance. Accordingly, the benefits may go beyond Hcy lowering and generate positive outcomes also independently of the direct effect on Hcy.

Thereafter, our micronutrient-supplemented patients enjoyed a double implantation rate and, although the study was not powered to detect differences in hard clinical outcomes, we also recorded a non-significantly larger (doubled) clinical pregnancy rate. This may mean that, besides an increased yield in blastocysts, also the quality of those blastocysts was intrinsically improved, which remains to be investigated.

Summarizing, follicular Hcy behaves as a reliable reporter of follicle/oocyte reproductive competence and it can be decreased with the supplementation of an array of micronutrients feeding the OCM. Such decrease associates to increased sensitivity to FSH, to an improved capacity of zygotes to develop in vitro to the blastocyst stage and in improved ability to implant, likely leading to increased chances of pregnancy. These clinical gains following a reduction of follicular Hcy further endorse the pathogenetic role of Hcy and the utility of testing it in follicular fluid.

The above decrease of follicular Hcy, as well the one reported by Schiuma et al. (2019) in blood, and the described clinical gains were achieved using physiological daily doses of the micronutrients of concern. This is of high clinical relevance.

First, as far as physiologic daily doses of micronutrients are concerned, this can be in theory achieved with dietary modifications, i.e., without recurring to a pharmaceutical supplement. The 400 μg of activated folates (5MTHF) and the 2.5 μg of activated B12 (methylcobalamin) provided by our supplement, which are likely the key substances for the effect, can easily be achieved by a balanced diet without engaging in complex dietary manipulations. Attention in avoiding contaminated foods (endocrine disruptors, heavy metals) would also help. Moreover, even if formulated supplements may be necessary, e.g., if exposures cannot be completely avoided or in case of very negative combinations of genetic variants, a healthy/varied background diet remains a mandatory requirement for a metabolic balance.

Second, similar results, both in reducing Hcy and in improving the clinical outcomes, have never been reached with plain folic acid supplementations, even when administered in huge doses. Asemi et al. (2014), working with PCOS women, recorded no effect on blood Hcy from 1 mg daily folic acid (2.5 fold our dose of 5MTHF) and a significant but partial reduction only for the dose of 5 mg per day (12.5 fold our dose of 5MTHF) [24]. The obvious explanation for these differences is the low ability of folic acid, which is an industrial surrogate, to substitute the natural forms of folates. Folic acid requires a double activation (reduction) by the enzyme dihydrofolate reductase (DHFR) to which it has very low affinity so that, at higher concentrations, it behaves as a competitive inhibitor of DHFR [25] and may result in an impairment of the whole pathway. Servy et al. [2018] clearly showed how shifting from folic acid to the natural form 5MTHF was enough to address a variety of complicated cases of couple infertility [26]. However, other physiopathologic mechanisms are in place. Besides being a metabolic reporter and a cellular poison, Hcy is also the starting substrate for several pathways moving toward 3 different directions (Fig. 1) and the key for metabolic compensation is its correct partitioning of Hcy among these 3 pathways. Forcing Hcy elimination with pharmacologic doses of folic acid will be therefore of low efficacy in those whose HHcy does not depend on failures in the folate pathway. Moreover, and even more relevant, the forced elimination of Hcy by the folate pathway in those not having that specific problem does further decrease Hcy availability as substrate for the other pathways. This jeopardizes cellular metabolism introducing new imbalances in spite of some “cosmetic” reduction of Hcy. In other words, and as far as a healthy pregnancy is the target, we do not simply need to lower Hcy, we need the metabolism to flow in all directions and this cannot be achieved with pharmacologic doses of single substances. Rather, as far as the individual genetic background is not known (and not metabolically understandable even if it was known), providing a balanced diet and/or a wider array of micronutrients at physiologic amounts might be the way to decrease Hcy without creating counter perturbations. The good clinical outcomes recorded in our patients strongly endorse this view.

The present study carries several limits and will need confirmations. It was of small size and not statistically powered. Moreover, we had not measured baseline fasting blood Hcy and cannot infer on the relationship between circulating and follicular Hcy, i.e., we cannot prove that they may have divergent behaviors. Moreover, we did not check for baseline folate and B12 status of our patients and cannot exclude imbalances between the groups in the pre-treatment status, which may happen is small size studies like the present one. Finally, we did not test our patients for signs of metabolic syndrome and for insulin resistance, both supposed to exert an effect of the fertility of PCOS patients and potentially modified by our micronutrients, which will need to be tested in duly designed studies.

On the other side, the present findings may have wider implications. We studied PCOS patients because some previous clinical data were available [13]; however, PCOS patients may not have a specific problem with Hcy metabolism [9] whereas it is well known that HHcy negatively affects human reproduction independently of the PCOS phenotype. In a recent large cohort study not restricted to PCOS, blood fasting Hcy was negatively associated with first trimester embryonic growth, which allowed the Authors to call for blood Hcy to be tested in both partners in every (intended) pregnancy [Rubini et al. 2022]. Similarly, it is extremely likely that follicular Hcy may act as a follicle quality marker also in non-PCOS infertile women and that a better control of intra-follicular Hcy would benefit any women suffering idiopathic infertility, which is to be confirmed by enlarged studies.

In conclusion, due to the high predictivity, low cost, easy access, and no added hazard to patients, we propose further investigations on follicular Hcy as a tool for oocyte-embryo selection in women undergoing COH for assisted reproduction purposes, whether or not affected by PCOS. This is of interest also because, as we show, follicular Hcy can be modulated by dietary manipulation, possibly even without recurring to the administration of pharmaceutical supplements. If dietary supplements are to be used for any reasons, given the availability of balanced supports able to achieve a metabolic balance in a physiologic way, we strongly discourage from treating infertile patients with HHcy, either in blood or in follicles, with pharmacologic doses of folic acid or any other single substances.
